# “Differential risk of hospitalization among single virus infections causing influenza‐like illnesses”

**DOI:** 10.1111/irv.12606

**Published:** 2018-10-16

**Authors:** Ana A. Ortiz‐Hernández, Katherine K. Nishimura, Daniel E. Noyola, Sarbelio Moreno‐Espinosa, Ana Gamiño, Arturo Galindo‐Fraga, Rafael Valdéz Vázquez, Martín Magaña Aquino, Alejandra Ramirez‐Venegas, Raydel Valdés Salgado, Diana Andrade‐Platas, Juliana Estevez‐Jimenéz, Guillermo M. Ruiz‐Palacios, Maria Lourdes Guerrero, John Beigel, Mary C. Smolskis, Sally Hunsberger, Laura Freimanis‐Hence, Beatriz Llamosas‐Gallardo, the Mexico Emerging Infectious Diseases Clinical Research Network (La Red)

**Affiliations:** ^1^ Instituto Nacional de Pediatria Ciudad de México México; ^2^ National Institute of Allergy and Infectious Diseases National Institutes of Health Bethesda Maryland; ^3^ Universidad Autonoma de San Luis Potosi San Luis Potosí México; ^4^ Hospital Infantil de México Dr. Federico Gómez Ciudad de México México; ^5^ Instituto Nacional de Ciencias Médicas y Nutrición Salvador Zubiran Ciudad de México México; ^6^ Hospital General Dr. Manuel Gea González Ciudad de México México; ^7^ Hospital Regional Dr. Ignacio Morones Prieto San Luis Potosí México; ^8^ Instituto Nacional de Enfermedades Respiratorias Ciudad de México México; ^9^ Westat Rockville Maryland; ^10^ Frederick National Laboratory for Cancer Research Leidos Biomedical Research, Inc. Frederick Maryland

**Keywords:** acute respiratory infection, hospitalization, influenza‐like illness, single virus

## Abstract

**Background:**

Acute respiratory infections are a major cause of morbidity in children and are often caused by viruses. However, the relative severity of illness associated with different viruses is unclear. The objective of this study was to evaluate the risk of hospitalization from different viruses in children presenting with an influenza‐like illness (ILI).

**Methods:**

Data from children 5 years old or younger participating in an ILI natural history study from April 2010 to March 2014 was analyzed. The adjusted odds ratio for hospitalization was estimated in children with infections caused by respiratory syncytial virus (RSV), metapneumovirus, bocavirus, parainfluenza viruses, rhinovirus/enterovirus, coronavirus, adenovirus, and influenza.

**Results:**

A total of 1486 children (408 outpatients and 1078 inpatients) were included in this analysis. At least one virus was detected in 1227 (82.6%) patients. The most frequent viruses detected as single pathogens were RSV (n = 286), rhinovirus/enterovirus (n = 251), parainfluenza viruses (n = 104), and influenza A or B (n = 99). After controlling for potential confounders (age, sex, recruitment site, days from symptom onset to enrollment, and underlying illnesses), children with RSV and metapneumovirus infections showed a greater likelihood of hospitalization than those infected by parainfluenza viruses (OR 2.7 and 1.9, respectively), rhinovirus/enterovirus (OR 3.1 and 2.1, respectively), coronaviruses (OR 4.9 and 3.4, respectively), adenovirus (OR 4.6 and 3.2, respectively), and influenza (OR 6.3 and 4.4, respectively).

**Conclusions:**

Children presenting with ILI caused by RSV and metapneumovirus were at greatest risk for hospitalization, while children with rhinovirus/enterovirus, parainfluenza, coronavirus, adenovirus, and influenza were at lower risk of hospitalization.

## 1 INTRODUCTION

Acute respiratory tract infection (ARI) represents the most frequent cause of outpatient visits to health care systems and hospitalization.^1^ ARI is a common illness in children, who experience an average of five to six ARI episodes every year with an increase during the second year of life.^2^ Lower respiratory tract infections (LRTI) are one of the main complications. Globally, LRTI is the third leading cause of years of life lost^3^ and is responsible for 16% of all deaths in children under 5 years.^4^ In 2015, 0.92 million children died of pneumonia; most of them were children <5 years old.^5^ Globally, the proportion of severe pneumonia episodes was 8.6% in 2000 and in 2010 an incidence of 11.5% was estimated for low‐and middle‐income countries.^6^ In Mexico, children less than 5 years of age account for up to 26.8% and 25.5% of all ARI and pneumonia cases, respectively.^7^

Many studies have sought to determine the viral etiology of ARI in children, and the importance of some viruses, such as influenza and respiratory syncytial virus (RSV), is well known.^8^, ^9^ Previous studies have detected one or more viruses in a high proportion of ILI cases, ranging from 76.6% of cases in physicians’ offices,^1^ 65% in outpatients,^10^, ^11^, ^12^ and between 39% and 60% in hospital settings.^11^, ^13^ In recent years, the sensitivity of diagnostic tests has improved and the number of detectable pathogens has increased.^14^ However, in Mexico and other Latin American countries, there is limited information regarding the prevalence and detection of viruses other than RSV and influenza as a cause of severe infections.^14^, ^15^, ^16^, ^17^

In the present study, we analyzed children 5 years old and younger recruited from the Mexican Emerging Infectious Diseases Clinical Research Network (La Red) ILI‐002 study. The objective of this study was to describe viruses detected in sick children (ambulatory and hospitalized) with ILI in Mexico, and to determine the contribution of these viruses to hospitalization risk.

## 2 MATERIAL AND METHODS

### 2.1 Study population

Data from this analysis were collected from individuals originally enrolled in the ILI‐002 study in the Mexico Emerging Infectious Disease Clinical Research Network (La Red) (Clinical Trials identifier NCT01418287), which recruited a prospective cohort of ILI patients year‐round from April 2010 to March 2014. Participants were recruited from five hospitals in Mexico City and one hospital in San Luis Potosi. Patients were eligible for participation if they met the study definition of ILI: at least one respiratory symptom (shortness of breath, postnasal drip, or cough) and one of the following general symptoms: fever ≥ 38°C or feverishness, malaise, headache, myalgia, or chest pain. Informed consent was obtained for all participants or parents/guardians, and the study was approved by each hospital's ethics committee. Complete details on the recruitment protocols and data collection have been published previously.^18^ For this subgroup analysis, we restricted the study to children who were 5 years old or younger with confirmed viral infections.

### 2.2 Study variables

The primary outcome of interest was admission to the hospital as a marker of a severe ILI. Since the emergency departments (ED) at the participating hospitals were often utilized by patients seeking both emergency and nonemergency care, patients who were in the ED for <24 hours were considered as outpatients. All deaths, regardless of hospitalization status, were considered to have severe ILI. All patients had follow‐up 14 days (by telephone call) and 28 days (clinic visit) after enrollment. Patients initially seen as outpatients who were later admitted to the hospital were included in the hospitalized group for this analysis.

Demographic information and medical histories were obtained from all eligible participants at the baseline study visit. A nasopharyngeal swab or nasal aspirate was collected at the time of recruitment from all patients. Viral pathogens were detected using multiplex real‐time PCR using the RespiFinder19 (April 2010 to May 2012) or RespiFinder 22 (previously RespiFinder Plus, PathoFinder BV, Maastricht, The Netherlands; June 2012 to March 2014); both tests detect the following viral pathogens: rhinovirus, RSV types A and B, influenza A and B, coronavirus (NL63, OC43, 229E), human metapneumovirus (hMPV), parainfluenza virus (PIV) types 1‐4, and adenovirus. RespiFinder 19 also included influenza H5N1, while RespiFinder 22 includes bocavirus, coronavirus HKU1, influenza A H1N1v, and enterovirus, but does not differentiate between rhinovirus and enterovirus. In addition, RespiFinder also detects four bacteria: *Bordetella pertussis*,* Chlamydophila pneumoniae*,* Legionella pneumophila*, and *Mycoplasma pneumoniae*. All samples that were originally tested with RespiFinder 19 were subsequently tested for bocavirus with primers specific for this virus. Because the aim of this study was to assess the severity of viral infections, patients in whom any of these bacterial pathogens were detected (either alone or in combination with viruses) were excluded from further analysis.

### 2.3 Statistical analysis

After excluding those with bacterial infections, we compared potential risk factors for severe ILI among those who were hospitalized vs those who were treated as outpatients. Univariate comparisons were evaluated using simple descriptive statistics. Differences in the prevalence in categorical variables were compared using chi‐square statistics, and differences in means in continuous variables were compared using *T* tests. Fisher's exact *P*‐values were reported for any categorical variable with cell counts less than five.

Logistic regression models were used to compare relative ILI severity associated with a given virus. Hospitalization was considered to be an indicator of severe ILI resulting from specific types of viral infections: RSV (types A or B), hMPV, bocavirus, PIV (types 1‐4), rhinovirus/enterovirus (RV/EV), coronavirus (NL63, OC43, 229E), adenovirus, and influenza (A or B). All models controlled for confounding by age, sex, recruitment site, days from symptom onset to enrollment, and presence of underlying illnesses. To reduce the uncertain contribution of effects due to possible interactions in multi‐virus infections, only patients with single virus infections were included in these models. The same model allowed for comparison of each virus to each of the other viruses and estimated whether the resulting odds ratio is significantly different from 1.0. Analyses were conducted using the Statistical Analysis System (SAS Institute, Cary, NC, USA) and R (R Foundation for Statistical Computing, Vienna, Austria).

## 3 RESULTS

From the original 5662 patients in the ILI‐002 study, there were 1550 children 5 years old or younger who had respiratory samples available for testing. A bacterial pathogen was detected in 64 of these children, and they were excluded from further analysis. Of the remaining 1486 patients, 408 (27.5%) were evaluated as outpatients and 1078 (72.5%) required admission to the hospital. Demographic and clinical characteristics of the study patients are described in Table 1. Children <1 year of age, children who had a longer duration between ILI symptom onset and arrival at the hospital, and those who had an underlying disorder (particularly congenital malformations) were more likely to be hospitalized.

**Table 1:** Demographic and clinical characteristics of children 5 y old or younger with ILI

	All (n = 1486)	Outpatient or ED < 24 h (n = 408)	Hospitalized (n = 1078)	*χ* ^2^ *P*‐value^a^
Sex				
Male	829 (55.8%)	225 (55.1%)	604 (56%)	0.80
Female	657 (44.2%)	183 (44.9%)	474 (44%)	
Age				
<1 y old	537 (36.1%)	82 (20.1%)	455 (42.2%)	<0.001
1‐2 y old	400 (26.9%)	109 (26.7%)	291 (27%)	
3‐5 y old	549 (36.9%)	217 (53.2%)	332 (30.8%)	
Days from symptom onset^b^				
0‐1 d ago	585 (39.4%)	248 (60.8%)	337 (31.3%)	<0.001
2‐3 d ago	389 (26.2%)	77 (18.9%)	312 (28.9%)	
4 or more days ago	509 (34.3%)	83 (20.3%)	426 (39.5%)	
Missing	3 (0.2%)	0 (0%)	3 (0.3%)	
Current tobacco smoke exposure				
No	1193 (80.3%)	328 (80.4%)	865 (80.2%)	0.99
Yes	293 (19.7%)	80 (19.6%)	213 (19.8%)	
Any comorbidity				
No	882 (59.4%)	322 (78.9%)	560 (51.9%)	<0.001
Yes	604 (40.6%)	86 (21.1%)	518 (48.1%)	
Viral infection				
No (0 viruses detected)	259 (17.4%)	82 (20.1%)	177 (16.4%)	0.11
Yes (1 or more viruses detected)	1227 (82.6%)	326 (79.9%)	901 (83.6%)	
Number of viral infections				
0	259 (17.4%)	82 (20.1%)	177 (16.4%)	0.04
1	972 (65.4%)	246 (60.3%)	726 (67.3%)	
2 or more	255 (17.2%)	80 (19.6%)	175 (16.2%)	

Chi‐square *P*‐values reflect tests that have omitted “missing” as a category. For any comparison with a cell with n < 5, a Fisher's Exact *P*‐value was reported.

Days between symptom onset and enrollment into the study (hospital admission).

At least one virus (either alone or in combination) was detected in 1227 (82.6%) patients. The frequency of detection of single viral infections were as follows: RSV (n = 286), RV/EV (n = 251); PIV (n = 104); influenza A or B (n = 99, with n = 73 having a single Influenza A infection, and n = 24 having a single Influenza B infection); hMPV(n = 97); coronavirus (n = 58); adenovirus (n = 45); and bocavirus (n = 32). The number of single virus infections and those in which each virus was detected in combination with another virus are shown in Table 2. Some viruses were detected as single infections more frequently than others; for instance, RSV and RV/EV were found as the single pathogen in most infections in which this virus was involved (78% and 68%, respectively), while bocavirus and adenovirus were more commonly found in the presence of other viruses (co‐infection rate of 60% and 56%, respectively).

**Table 2:** Viral coinfections in children 5 y old or younger with ILI

	RSV^a^	Metapneumovirus	Bocavirus	Parainfluenza^b^	Rhinovirus	Coronavirus^c^	Adenovirus	Influenza A or B
RSV	(286)							
Metapneumovirus	2	(97)						
Bocavirus	5	8	(32)					
Parainfluenza	14	8	7	(104)				
Rhinovirus	33	9	20	22	(251)			
Coronavirus	14	10	2	12	12	(58)		
Adenovirus	9	8	9	5	21	6	(45)	
Influenza A or B	12	10	8	3	13	14	6	(99)

Numbers in parentheses are counts for single infections. Coinfection counts include 2‐virus infections and 3 or more virus infections. Three viruses were detected simultaneously in 21 patients in 14 different combinations; among these, RV/EV were detected in 10, bocavirus in 9, coronavirus in 8, influenza in 8, metapneumovirus in 8, parainfluenza in 8, RSV in 6, and adenovirus in 6. There was one patient in whom 4 viruses were detected: RSV, influenza, coronavirus, and bocavirus.

a Includes RSV A and B.

b Includes parainfluenza types 1, 2, 3, and 4.

c Includes coronavirus 229E, NL63, OC43, and HKU1.

**Figure 1:**
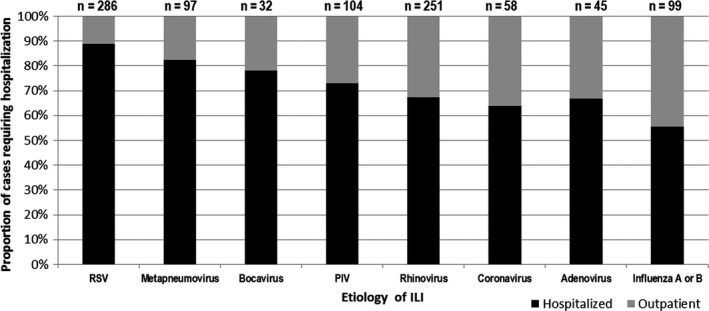
Proportion of patients ≤5 y of age with ILI that required hospitalization or were treated as outpatients, according to etiology. Only children with single viral infections are included

The majority of patients in this cohort were hospitalized. However, there were notable differences of hospitalized proportions between the different viruses. Among those with single virus infections, the proportion of patients who were treated as outpatients or required hospitalization varied greatly depending on the underlying viral infection (Figure 1). For instance, almost 90% of patients with RSV infection were hospitalized, while among patients with influenza infection, this proportion was 55%.

**Figure 2:**
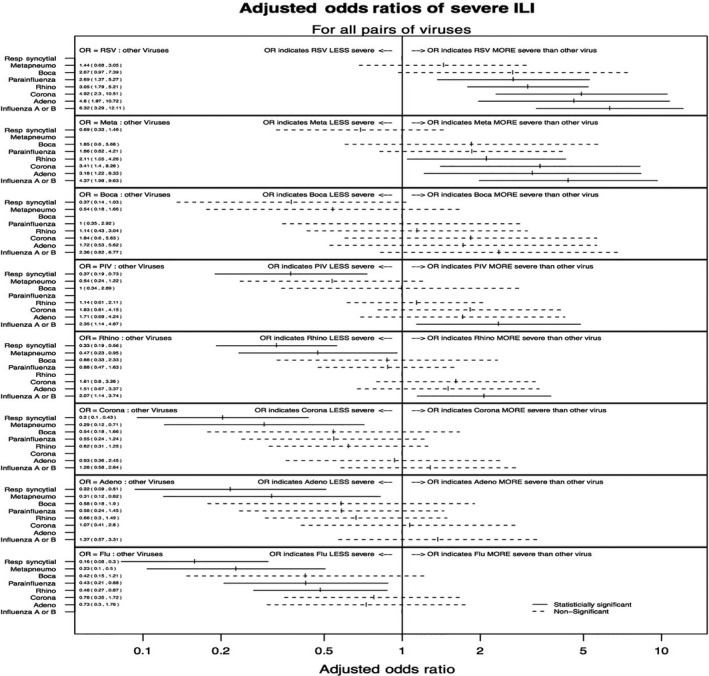
Pairwise comparisons for the likelihood of requiring hospitalization in children ≤5 y of age with ILI caused by different viruses. Each panel shows the comparison between a specific virus and each of the other viruses included in the study. The adjusted odds ratios (taking into account age, sex, days from onset of symptoms, presence of underlying illness, and study site) are shown for comparisons between each pair of viruses. Statistically significant differences are shown in solid lines; nonsignificant comparisons are shown in dotted lines

When we used logistic regression models to compare the relative ILI severity between viruses, while controlling for sources of confounding identified in univariate tests, patients with ILI caused by RSV showed a greater likelihood of hospitalization than those infected by most other viruses, except hMPV and bocavirus (Figure 2). Patients with hMPV infection had a greater likelihood of hospitalization than those with infections caused by RV/EV (OR = 2.11 [95% CI 1.05‐4.26]), coronavirus (OR = 3.41 [95% CI 1.40‐8.26]), adenovirus (OR = 3.18 [95% CI 1.22‐9.63]), and influenza viruses (OR = 4.37 [95% CI 1.98‐9.63]). Patients suffering from PIV infections were more likely to require hospitalization than those with influenza infection (OR = 2.35 [95% CI 1.14‐4.87]), but were less likely to require hospitalization than those with RSV infection (OR = 0.37 [95% CI 0.19‐0.73]). Among patients with infections caused by coronavirus, RV/EV, adenovirus, and influenza viruses, many associations were not statistically significant, however, all of them were less likely to require hospitalization when compared to RSV and hMPV.

## 4 DISCUSSION

The advance in diagnostic methods with increased sensitivity to detect respiratory viruses in recent years requires continued research to understand the prevalence of these viruses in children and to compare their ability to cause life‐threatening illness as single infections. Lower respiratory tract infections including pneumonia have increased rates that coincide when respiratory viruses are more prevalent^19^ Consistent with previous studies, we found that RSV was highly associated with hospitalizations, appearing to cause worse ILI outcomes when compared to single PIV, RV/EV, coronavirus, adenovirus, and influenza infections. Metapneumovirus (hMPV), though less common than RSV (9.7% and 24.7%, respectively) was more prevalent in our study than others had previously reported (4‐5.5%).^20^ hMPV was significantly associated with hospitalization; however, its ORs were generally smaller than those of RSV. We also found that a delayed visit to the doctor was more common among patients that required hospitalization. This finding underscores the importance of educating the public about seeking medical care soon after symptom onset to prevent more severe ILI outcomes. The higher relative severity of RSV and hMPV infections probably reflects their greater capacity to cause lower respiratory tract disease in young infants. Longitudinal studies have shown that 22.4% of children suffer RSV‐associated lower respiratory infection during the first year of life^21^ Wheezing has been reported in 10% of children with hMPV infections^22^ and is also associated to hypoxia and fever^20^

While the prevalence of influenza infections was moderately high in this study population, with 10.4% of children having a detectable strain of influenza, these infections appear least likely to result in ILI‐associated hospitalization. A reduction in the proportion of influenza‐associated hospitalizations and mortality in Mexican children <5 years of age was reported after the inclusion of the influenza vaccine for routine immunization in 2004.^23^, ^24^ While some information about influenza vaccination was collected during recruitment into this study, the data were not detailed enough to thoroughly investigate the impact of vaccination on ILI‐associated hospitalizations. Coinfections with viral (influenza and RSV) and bacterial (*Streptococcus pneumoniae* and *Streptococcus aureus*) have previously been reported to cause severe ILI outcomes.^25^, ^26^ While we would have liked to investigate the relationship between viral and bacterial coinfections, the number of children in our study with these types of infections was too small to analyze. When influenza virus is circulating and the incidence of severe lower respiratory tract infections begins to increase, clinicians should consider empirical treatment for influenza and antibiotic therapy.

Of all eight types of viruses, bocavirus was the one least frequently detected (5.3%). Though several of the bocavirus ORs were greater than 1.0, we were likely underpowered to determine whether these associations truly exist. Interestingly, bocavirus was the virus that presented the highest proportion of coinfections, a finding similar to that of other reports where it has been reported that up to 75% of the bocavirus infections are co‐infections with one or more viruses,^27^ a prolonged viral shedding up to 4.5 months in hospitalized patients has been described^28^ and in outpatients about 75 days^29^ this will explains the detection in asymptomatic patients.^30^ Though we would have liked to assess the role of bocavirus coinfections in severe ILI, there were not enough individuals to make a valid comparison. Because few studies of bocavirus infections have included control groups, the disease burden associated to this virus is still unclear.^31^ Thus, additional research is required to define the pathogenic potential of this virus.

Our follow‐up of all participants 2 and 4 weeks after enrollment into the study helped ensure that patient outcomes were recorded correctly, reducing the amount of outcome misclassification. Additionally, our prospective study design and the expanded panel of the RespiFinder 22 kit allowed us to perform an analysis of ILI outcomes among children with different single virus infections, providing unique insight into the relative severity of these infections.

A limitation of the study is that our sample is restricted to patients meeting an ILI case definition; thus, our results might not be generalized to all acute respiratory infections. Nevertheless, the ILI definition that we used allowed for the inclusion of a broad range of clinical presentations, and, therefore, it is likely that this data might be of help for assessment of patients that seek acute medical care. We acknowledge that the decision to seek medical attention in a hospital (rather than in a primary care clinic) could be related to the perceived severity of the illness, potentially biasing our study population. As our study was primarily hospital based, the absolute rates of hospitalized vs outpatients are likely skewed towards those that present to a hospital for care of the ILI. However, we believe that the place of choice for medical attention would not confound the risk for hospitalization among those with severe ILI, regardless of the infectious agent should not influence the relative proportions of hospitalization between viruses.

As mentioned previously, there were a number of children with viral coinfections. Although we suspect that each combination of viruses could result in specific virus‐virus interactions, we did not have a sample large enough to examine this matter further. Finally, findings for RV/EV might be limited by the fact that the RespiFinder PCR assay does not distinguish between rhinovirus and enterovirus, and differences between these picornaviruses could not be elucidated any further.

In summary, the availability of prospectively recorded data in this large cohort of patients provided us with the opportunity to describe the viral etiology and ILI severity in Mexican children. Our results indicate that among children with ILI, those with RSV and hMPV infections were at greatest risk for hospitalization, while children with RV/EV, PIV, coronavirus, adenovirus, and influenza were at lower risk. The observed differences in the likelihood of hospitalization between children with diverse etiologies support the need for closer observation in those with infections caused by RSV or hMPV.
